# Cobalt Oxide Nanoparticles/Graphene/Ionic Liquid Crystal Modified Carbon Paste Electrochemical Sensor for Ultra-sensitive Determination of a Narcotic Drug

**DOI:** 10.15171/apb.2019.014

**Published:** 2018-02-21

**Authors:** Nada Farouk Atta, Ahmed Galal, Ekram Hamdy El-Ads, Samar Hamed Hassan

**Affiliations:** ^1^Department of Chemistry, Faculty of Science, Cairo University, 12613, Giza, Egypt.; ^2^Forensic Chemistry Laboratory, Medico Legal Department, Ministry of Justice, Egypt.

**Keywords:** Morphine, Dopamine, Cobalt oxide nanoparticles, Graphene, Ionic liquid crystal

## Abstract

***Purpose:*** Drug-abuse, namely morphine (MO) affects the metabolism of neurotransmitters such as dopamine (DA). Therefore, it is crucial to devise a sensitive sensing technique to simultaneously determine both compounds in real samples.

***Methods:*** The fabrication of the sensor is based on in situ modification of a carbon paste (CP) electrode with cobalt oxide nanoparticles, graphene, and ionic liquid crystal in presence of sodium dodecyl sulfate; CoGILCCP-SDS. The modified sensor is characterized using scanning electron microscopy, electrochemical impedance spectroscopy and voltammetry measurements.

***Results:*** Electron transfer kinetics and analytical performance of the proposed sensor were enhanced due to the synergistic role of all the modifiers. The simultaneous determination of MO and DA achieved low detection limits of 0.54 nmol L−1 and 0.25 nmol L−1, respectively. Besides, a carbon-based electrochemical sensor is fabricated for the nano-molar determination of MO in real samples and formulations. The sensor showed fouling resistance and anti-interference ability in presence of other species in human fluids. The real sample analysis of MO was successfully achieved with good recovery results in urine samples and pharmaceutical tablets. Linear dynamic range, sensitivity, detection limit and quantification limit of MO in urine were 5 nmol L−1 to 0.6 μmol L−1, 6.19 μA/μmol L-1, 0.484 nmol L−1 and 1.61 nmol L−1, respectively.

***Conclusion:*** This sensor has great ability to be extended for electrochemical applications in assaying of many drugs.

## Introduction

Morphine (MO), the main ingredient of opium poppy, is mainly responsible of poisoning and death cases particularly for those with a drug addiction and abuse history. It is frequently used as a relief of moderate to severe pains. Overdoses of MO may be fatal with symptoms including slow heart and breathing rate, weakness of muscles, cold, severe sleepiness and syncope. Also, MO uptake leads to some side effects namely; dizziness, stomach pain, headache, nausea, vomiting, anxiety and mild itching.^1-4^ In forensic cases, existence of MO in blood or urine is an index of heroin uptake.^5^ So, it is very vital to develop methods for sensitive and selective determination of MO. Many methods have been utilized for this purpose like ultraviolet spectroscopy,^6^ chromatography-tandem spectrometry,^7^ thin layer chromatography,^8^ radioimmunoassay,^9^ surface plasma resonance,^10^ and electrochemical methods.^11-18^

Carbon paste (CP) electrodes have found great era of applications in electrochemical studies and electroanalysis because of their facile preparation, low cost, suitability for a wide range of different sensing applications and surface renewability.^19,20^ Graphene, merged in the world of nanotechnology and discovered by Andre Geim in 2004, is a 2D honey comb-like structure containing sp^2^ carbon atoms. Graphene, in the thickness of one atom, has attracted great awareness due to its fascinating electronic, mechanical and thermal characteristics. Graphene, the mother of carbon materials, showed unique characteristics such as large theoretical surface area of about 2620 m^2^.g^-1^, thermal, chemical and electrical conductivity, and low cost through its production compared to other carbon materials. More properties were chemical stability, impermeability to gases, high mobility of charges (both electrons and holes), presence of large amount of edge planes/defects, charge-transfer characteristics and feasibility of functionalization.^19,21,22^ Also, graphene has a set of oxygenated species at the edges of its sheets acting as specific surface functional groups.^23^ Graphene and graphene based composites have found great impact in different applications like sensors, catalysis, electronics, energy storage and conversion, water purification, etc.^21,22,24^ Furthermore, graphene based composites have been widely used as electrochemical sensors.^18,25-27^

On the other hand, graphene/metal or metal oxides hybrid showed the synergistic effect of graphene as a conductive catalyst support with large specific surface area and metal or metal oxide nanoparticles as electrical conductive antennae.^21,22^ Nanomaterials have been widely used to increase the surface area of different surfaces, improve the electron transfer rate and electrode processes kinetics, provide large edge plane/basal plane ratios and act as molecular recognition elements to enhance the sensor sensitivity.^22,28^ Cobalt oxide (Co_3_O_4_), p-type semiconductor, has a spinel crystal structure (Co^2+^ (Co_2_^3+^)O_4_) where Co^3+^ and Co^2+^ ions are coordinated with oxygen ions in an octahedral and tetrahedral structure, respectively.^29,30^ Cobalt oxide nanoparticles have been the focus of many researchers owing to their biocompatibility, large specific surface area, environmentally friendly nature, chemical stability, electrocatalytic activity, excellent conductivity, antifouling ability, low cost, and availability.^22,29-34^ Cobalt oxide nanostructures modified electrodes can offer more active sites for the reaction and its porous structure allows the adsorption and diffusion of electroactive species.^30^ Therefore, cobalt oxide modified electrodes were found versatile in different applications of heterogeneous catalysis,^35^ magneto-resistive devices,^36^ electrochromic thin films,^37^ fuel cells, sensors, biomaterials, Li-ion batteries and supercapacitors.^38,29-34^

Ionic liquids (ILs) modified electrodes have gained much interest in different applications owing to the featured characteristics of ILs such as high polarity, high thermal stability, high viscosity, intrinsic conductivity and wide potential window.^39-41^ Ionic liquid crystals (ILCs), combining the unique properties of ILs and liquid crystals, showed superior ionic conductivity resulting in enhanced analytical performance.^42^ ILC modified electrodes have been recently used for determination of different drugs.^42,43^ Moreover, the application of surfactants in different fields of electrochemistry has proven to alter the electrode/solution interface and affect the electrochemical processes of electroactive species.^44,45^

Herein, we introduce a sensitive nanocomposite for determination of MO in human urine and pharmaceutical formulations with good precision and accuracy. The nanocomposite is fabricated by in-situ modification of CP electrode with cobalt oxide nanoparticles, graphene, and ILC for the effective determination of MO and dopamine (DA) in presence of sodium dodecyl sulfate (SDS); CoGILCCP-SDS. The influence of possible interferences in human fluids on the electrochemical signal of MO was illustrated. Furthermore, figures of merit for the sensor were investigated.

## Materials and Methods

All chemicals were used as received without further purification. MO sulfate was supplied by Forensic Chemistry Laboratory, Medico Legal Department, Ministry of Justice, Cairo, Egypt. Graphite powder (<20 µm, synthetic), paraffin oil, ILC (1-butyl-1-methyl piperidinium hexafluorophosphate), and ionic liquids (1-n-hexyl-3-methylimidazolium tetrafluoroborate) (IL1) and (1-butyl-4-methylpyridinium tetrafluoroborate) (IL2) were purchased from Aldrich Chem. Co. (Milwaukee, WI. USA). Sulfuric acid, hydrazine hydrate, SDS, cobalt oxide nanoparticles (Co_3_O_4_, <50 nm nano-powder), terazosin hydrochloride (TZ), ascorbic acid (AA), DA, potassium phosphate (mono, di-basic salts) and potassium hydroxide were purchased from Sigma-Aldrich. Phosphate buffer solution (PBS) (1.0 mol L^-1^ K_2_HPO_4_ and 1.0 mol L^-1^ KH_2_PO_4_) of pH 2-11 was used as the supporting electrolyte. The pH was adjusted using suitable amounts of 0.1 mol L^-1^ H_3_PO_4_ and 0.1 mol L^-1^ KOH. All solutions were prepared using double distilled water.

### Electrochemical cell and equipment

A three-electrode/one-compartment glass cell was used for electrochemical studies. The working and auxiliary electrodes were CP electrode and a 10 cm long/2.0 mm diameter Pt wire, respectively. All the potentials in the electrochemical studies were referenced to Ag/AgCl (4 mol L^-1^ KCl saturated with AgCl) electrode. All experiments were performed at 25°C ± 0.2°C. The electrochemical characterization of the different modified electrodes was achieved using a BAS-100B electrochemical analyzer (Bioanalytical Systems, BAS, West Lafayette, USA). The scanning electron micrographs of the different films were achieved via Quanta FEG 250 instrument (accelerating voltage was 20 keV).

### Preparation of graphene

Graphene oxide (GO) was prepared via modified Hummers and Offeman method. In order to prepare graphene “G”, GO was reduced using hydrazine hydrate under microwave irradiation.^18^

### Preparation of different modified surfaces

CP was prepared by mixing 0.5 g of graphite powder with 0.3 mL of paraffin oil in a glass mortar. CP was packed into the hole of the electrode body and smoothed on a filter paper until its shiny appearance.

To prepare CoGILCCP, a mixture of 25 % (w/w) ILC, 15 % (w/w) G, 5 % (w/w) Co_3_O_4_ and 55 % (w/w) graphite powder (optimized) was mixed with 0.25 mL of paraffin oil in a mortar with a pestle till a homogenous paste was obtained. Then, the resulting paste was packed firmly in the hole of the electrode body and the electrode was rinsed with water and dried in air at room temperature. The two other ionic liquids modified CP electrodes; (CoGIL1CP) and (CoGIL2CP), were prepared by the same way but using 1 % (w/w) ILs with respect to graphite powder and keeping the ratio of cobalt oxide constant. The modification of the different electrodes surfaces with SDS was accomplished by the addition of 20 µL from a stock of SDS (0.1 molL^-1^).

### Preparation of solutions for urine and tablets analysis

The proposed sensor was utilized in real sample analysis by direct analysis of human urine samples spiked with MO. Urine sample used for detection was diluted 300 times with 0.1 mol L^-1^ PBS/pH 7.4 to reduce the matrix effect of real samples.^43^ MO was dissolved in 0.1 mol L^-1^ PBS/pH 7.40 to prepare a 1 mmol L^-1^ stock solution. Standard additions were carried out from the MO stock solution in 15 mL of diluted urine.

Determination of MO in its pharmaceutical formulation was performed simply without any extraction steps or sample pretreatment. One tablet of MO (containing 30 mg MO) was weighed and crushed into fine powder. The fine powder was dissolved in 0.1 mol L^-1^ PBS/pH 7.40 to prepare a stock solution of MO (1 mmol L^-1^).

### Statistical analysis

Standard deviations, relative standard deviations (RDSs) and all other calculations were performed using Excel Spreadsheet software. The data were calculated following the “IUPAC” recommendations.

## Results and Discussion

### Electrochemical oxidation of morphine at different modified electrodes

**Figure 1:**
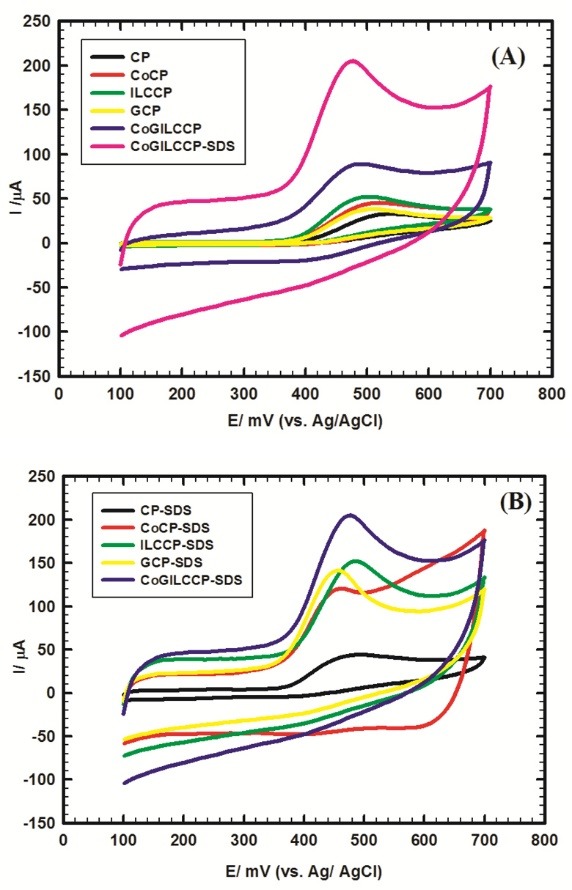
(A) Cyclic voltammograms (CVs) of 1 mmol L^-1^ morphine (MO)/0.1 mol L^-1^ phosphate buffer solution (PBS)/pH 7.40 at CP, CoCP, ILCCP, GCP, CoGILCCP and CoGILCCP-SDS. (B) Cyclic voltammograms (CVs) of 1 mmol L^-1^ morphine (MO)/0.1 mol L^-1^ phosphate buffer solution (PBS)/pH 7.40 at CP-SDS, CoCP-SDS, ILCC-SDS, GCP-SDS, and CoGILCCP-SDS, scan rate 50 mV L^-1^.

The electrochemical behavior of 1 mmol L^-1^ MO/0.1 mol L^-1^ PBS/ pH 7.4 was examined using cyclic voltammetry at different working electrodes; CP, GCP, CoCP, ILCCP and CoGILCCP and comparing it with the corresponding cases in presence of SDS (CP-SDS, GCP-SDS, CoCP-SDS, ILCCP-SDS and CoGILCCP-SDS) as shown in Figures (1A and 1B). The values of oxidation potential and current were summarized in Table 1. MO exhibited an irreversible oxidation behavior as mentioned in literature.^11-18^ A flagging response was obtained at CP due to the electrode fouling effect. While a slight increase in the anodic peak current with broad oxidation peak and high overpotential was obtained upon individually modifying CP with graphene, cobalt oxide nanoparticles or ILC (GCP, CoCP or ILCCP). The collective inclusion of graphene, ILC and cobalt oxide nanoparticles (CoGILCCP) resulted in higher current response and lower overpotential compared to the single effect of each modifier. Integrated effect was achieved by the individual modifiers reflecting the role presented by each one. ILC showed spontaneous orientation ordering and enhanced ionic conductivity owing to the stability of the ionic units in the mesophases by electrostatic interactions and ion-ion stacking. ILC with its ionic conductance can provide ionic paths toward the electrode surface.^44,45^ Graphene exhibited distinctive electronic structure with great electronic states density, high specific surface area, electrical conductivity, high charges mobility, chemical stability and existence of edge-plane defect sites acting as active sites for analytes. In addition, graphene with its unique 2D structure showed more uniform allocation of electrochemically active sites compared to graphite. All these characteristics resulted in the facilitation of charge transfer kinetics in terms of enhanced current response.^19,21-23^ Cobalt oxide nanoparticles showed large specific surface area, biocompatibility, good electrocatalytic activity, chemical stability and excellent conductivity resulting in charge transfer facilitation.^22,30-34^

**Table 1:** Summary of cyclic voltammogram (CV) results obtained at different modified electrodes toward 1 mmol L^-1^ morphine (MO)/0.1 mol L^-1^ phosphate buffer solution (PBS)/pH 7.40, scan rate 50 mV L^-1^

**Electrode**	**E**_pa_ ** / mV**	**I**_pa_ **/ µA**	**D**_app_ × 10^-5^ / cm^-2^ s^-1^
CP	535	32.6	0.489
GCP	505	37.0	0.630
CoCP	510	44.8	0.924
ILCCP	500	50.8	1.19
CoGILCCP	487	69.2	2.21
CP-SDS	487	40.1	0.741
GCP-SDS	457	115	6.08
CoCP-SDS	461	98.7	4.49
ILCCP-SDS	484	113	5.88
CoGILCCP-SDS	**478**	**173**	13.8
CoGIL1CP-SDS	497	76	2.66
CoGIL2CP-SDS	510	60	1.66
GILCCP	468	62.6	1.81
CoILCCP	481	66.1	2.01
CoGCP	480	56.8	1.49
GILCCP-SDS	492	118	6.41
CoILCCP-SDS	462	128	7.55
CoGCP-SDS	483	104	4.98
CoGILCCP-SDS	**478**	**173**	**13.8**

Upon the addition of 20 µL of 0.1 mol L^-1^ SDS, higher anodic peak current was obtained at CoGILCCP-SDS compared to CoGILCCP demonstrating the role of SDS. The current response of CoGILCCP-SDS was still much higher than that obtained at CP-SDS, GCP-SDS, CoCP-SDS and ILCCP-SDS indicating the catalytic behavior of the proposed composite. Moreover, the proposed sensor showed a well-defined sharp peak with high current response (Table 1) compared to the other studied modified electrodes; CoILCCP-SDS, GILCCP-SDS and CoGCP-SDS (Supplementary file 1).

SDS reinforced the preconcentration/accumulation of MO cations at the electrode surface. Besides, SDS adsorption over the electrode surface facilitated the electron transfer process, improved the current signal of MO and reduced the overpotential due to Ohmic-drop. Therefore, combining the role introduced by each modifier; ILC, graphene, cobalt oxide and SDS, resulted in the characteristic performance of the proposed nanocomposite.

### Catalytic activity of ILC versus other ILs in sensor design

The introduction of ILC in the sensor design showed featured performance and its role is elucidated by comparing the response of other ILs like (1-n-hexyl-3-methylimidazolium tetrafluoro-borate (IL1)) and (1-butyl-4-methylpyridinium tetrafluoro-borate (IL2)) towards the electro-catalytic oxidation of morphine. Supplementary file 2 showed the electrochemical response of 1 mmol L^-1^ MO/0.1 mol L^-1^ PBS/ pH 7.4 at CoGILCCP-SDS, CoGIL1CP-SDS and CoGIL2CP-SDS. Higher current response and lower oxidation potential were achieved at CoGILCCP-SDS compared to other electrodes (Table 1). This result confirmed the important role of ILC in improving the performance of the proposed electrode owing to its excellent ionic conductivity.^44,45^

### Morphology study

**Figure 2:**
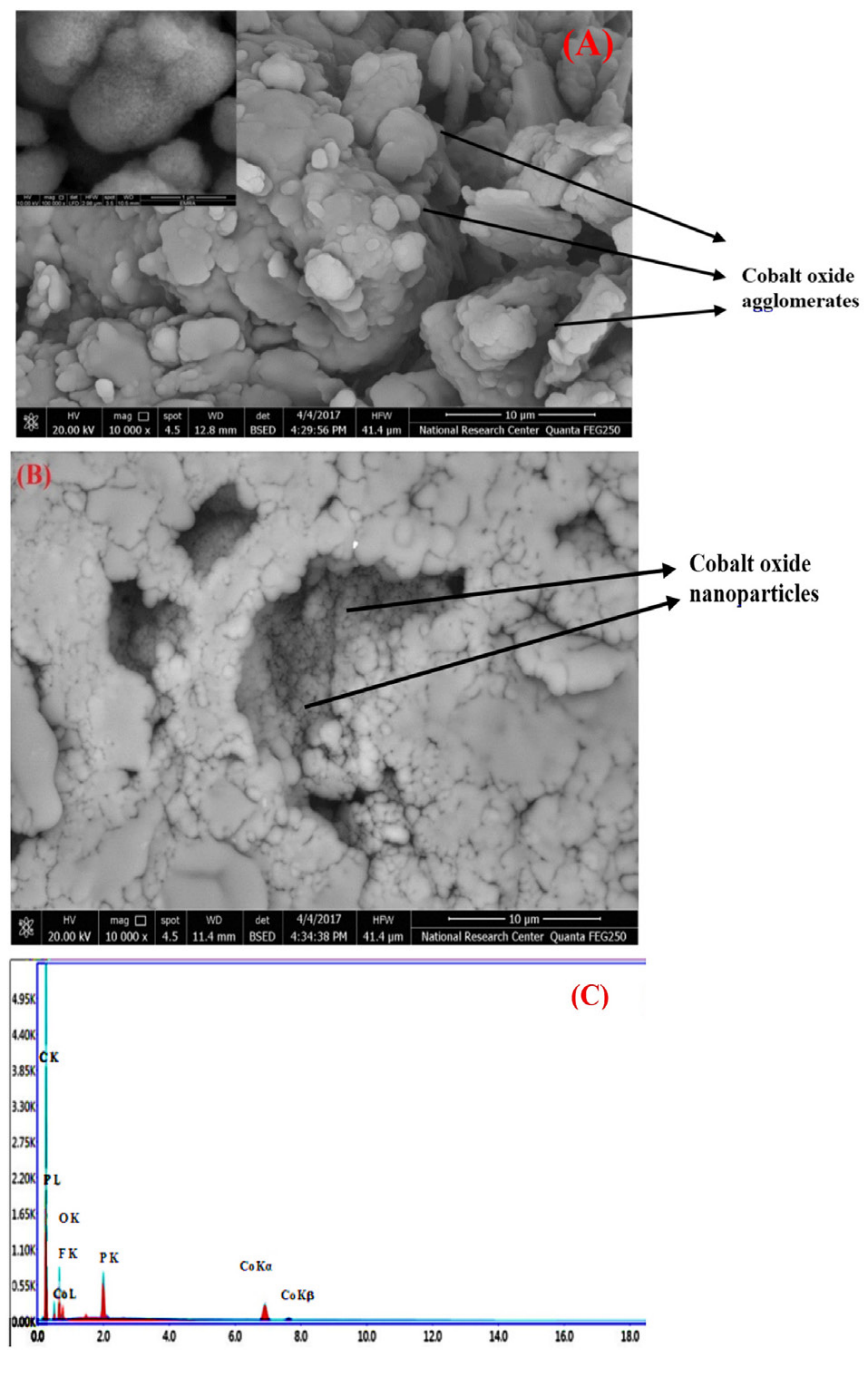
Scanning electron micrograph (SEM) images of **(A)** CoGILCCP (inset: high magnification; ×10^5^ times (for Co-nanoparticles agglomerates)) and **(B)** CoGILCCP-SDS; **(C)** EDAX of CoGILCCP.

The catalytic response of the proposed electrode towards MO oxidation can be explained in accordance to the surface morphology and the dispersion of different modifiers over the electrode surface. The SEM of CoGILCCP and CoGILCCP-SDS modified electrodes were shown in Figure 2A and 2B , respectively. The SEM of bare CP showed separated graphite flakes (Figure not shown). Figure 2A showed SEM of CoGILCCP electrode surface where cobalt oxide nanoparticles were dispersed between the graphite flakes and graphene sheets combined with ILC. Figure 2B showed different morphology upon the addition of SDS where a spongy film was formed over the surface supporting the preconcentration of MO at the electrode surface. Figure 2C showed the EDAX of CoGILCCP confirming the presence of cobalt oxide nanoparticles inside the paste.

### Electrochemical impedance spectroscopy

**Figure 3:**
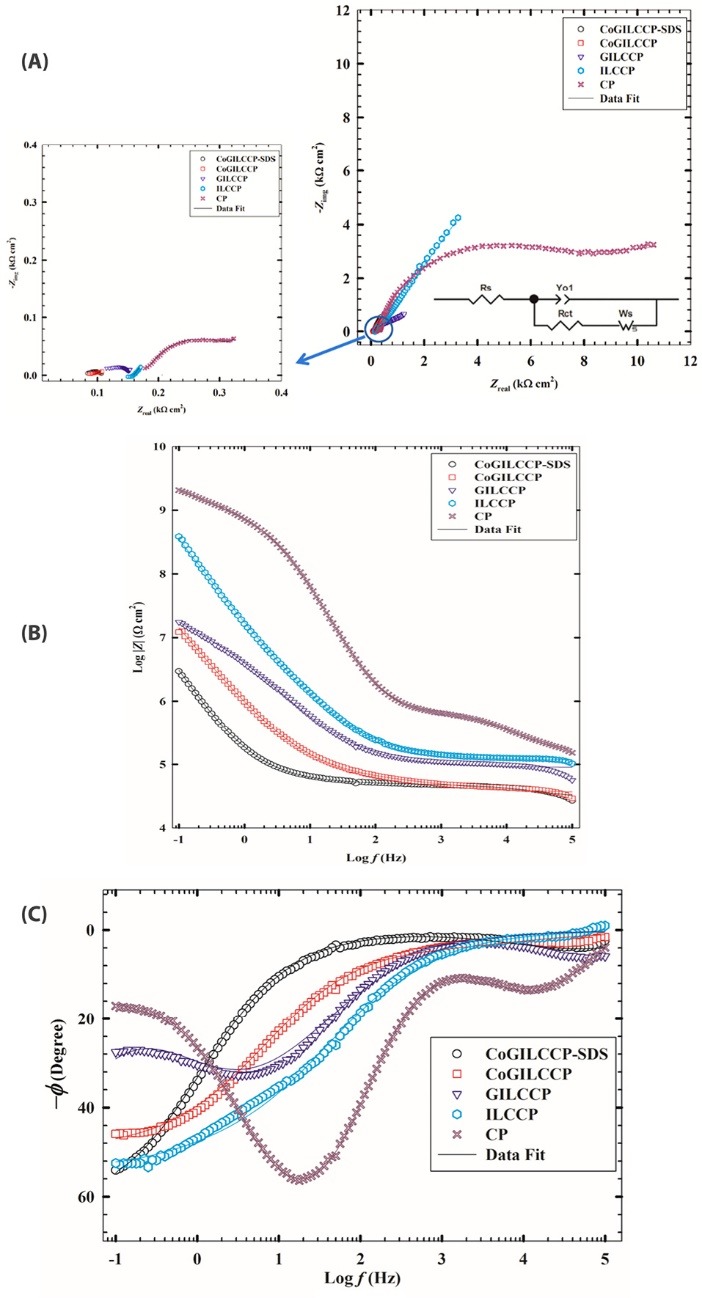
Typical impedance spectra presented in the form of Nyquist plots (A) (Inset: Equivalent circuit used in the fit procedure of the impedance spectra) and Bod plots (B&C) for CP (**×**), ILCCP (**ο**), GILCCP (‏֜∇), CoGILCCP (□) and CoGILCCP-SDS (**ο**). (Symbols and solid lines represent the experimental measurements and the data fitting, respectively).

Since the major contribution to the enhancement of charge transfer is attributed to surface rearrangements, Electrochemical impedance spectroscopy (EIS) helps understanding the interfacial properties of the modified electrodes surfaces. Moreover, EIS gives accurate information about the kinetics and mechanisms of different electrochemical systems including sensors. EIS experiments were performed in 1 mmol L^-1^ MO/0.1 mol L^-1^ PBS/pH 7.4 at an AC frequency in the range of 0.1 Hz to 100 kHz at the corresponding oxidation potential of MO at CP, ILCCP, GILCCP, CoGILCCP and CoGILCCP-SDS electrodes where it reflects the charge transfer kinetics upon the stepwise modification of the CP electrode. The impedance spectra in the form of Nyquist and Bode plots were shown in Figure 3A, 3B & 3C, respectively. The software used for fitting EIS data was provided with the instrument. The equivalent circuit used for fitting the EIS data was given in the inset of Figure 3A. In this circuit, *R*_s_ is the solution resistance, and *R*_ct_ is the charge transfer resistance (that is affected by changes at the interface). Capacitors in EIS experiments do not behave ideally; instead they are represented by constant phase elements. Constant phase element (*Y*_o1_) represents surface roughness and inhomogeneity of reaction rate and (n < 1) is its corresponding exponent. *W*_s_ is the Warburg impedance due to charge diffusion from solution bulk to electrode surface. The fitting data were represented in solid lines and there was a good agreement between the fitting and experimental data. Figure 3A showed that a quasi-semicircle portion with large diameter corresponding to the charge transfer resistance was obtained at higher frequency region in case of CP reflecting an electron transfer controlled process. While a linear portion was obtained at lower frequencies at CP corresponding to a diffusion controlled process. The semicircle decreased significantly in all the other modified electrodes in the order of ILCCP > GILCCP > CoGILCCP > CoGILCCP-SDS manifesting relatively lower charge transfer resistance and fast charge transfer kinetics upon modification of CP electrode. From the Nyquist and Bode plots, it was shown that there is a noticed decrease in the total impedance values upon modification assuring the distinct catalytic performance of the CoGILCCP-SDS electrode. The fitting data corresponding to Figure 3 were summarized in Table 2. An important feature in the data is the decrease in the value of *R*_ct_ that ascertains the increase in charge conductance upon ILC inclusion and due to the increase in the metallic character imparted by the presence of nanostructures in the film. Furthermore, the CoGILCCP-SDS electrode showed increased value of the interfacial capacitance component *Y*_o1_ compared to the CP due to an increase in ionic accumulation at the surface of the modified electrode.

**Table 2:** Electrochemical impedance spectroscopy (EIS) fitting data corresponding to Figure 3

	**CP**	**ILCCP**	**GILCCP**	**COGILCCP**	**COGILCCPSDS**
R_s_ (Ω cm^2^)	179	159	141	94.3	82.0
R_ct_ (kΩ cm^2^)	7.81	5.71	1.43	0.0371	0.0273
Y_o1_ (×10^-4^) (n) (S. s^n^)	0.101 0.876	2.38 0.902	3.62 0.806	5.39 0.877	6.18 0.997
W_s_ (kΩ cm^2^)	20.5	12.1	18.90	1.46	0.410
c^2^ Weighed S(x)^2^	2.28 × 10^-3^ 5.30 × 10^-1^	1.86 × 10^-3^ 4.41 × 10^-1^	1.17 × 10^-3^ 2.78 × 10^-1^	1.56 × 10^-3^ 3.71 × 10^-1^	1.24 × 10^-3^ 2.91 × 10^-1^

### Influence of operational parameters

#### Influence of scan rate

The electrochemical response of the proposed nanocomposite upon the application of different scan rates in1 mmol L^-1^ MO/0.1 mol L^-1^ PBS/pH 7.4 was examined. Inset of Supplementary file3 showed the cyclic voltammograms of 1 mmol L^-1^ MO/0.1 mol L^-1^ PBS/pH 7.4 at CoGILCCP-SDS at different scan rates from 10 to 100 mV L^-1^. Upon varying the scan rate from 10 to 100 mV L^-1^, the anodic current signal of MO increased and the oxidation potential was shifted to more positive values confirming the irreversible behavior of MO oxidation.^13-15,18,41^ A linear relationship was obtained between the anodic peak current of MO and the square root of scan rate (Supplementary file 3). This linear relation confirmed that the electrochemical oxidation of MO at CoGILCCP-SDS is under diffusion controlled.^11,12,14,15,18,41^

The linear regression equation could be fitted to (Eq. 1);

(1) Ip(A)=1.59×10-3 v1/2 (V/s)1/2−(−1.19×10−4)(Correlation coefficient is:  R2=0.995)

Randles-Sevcik equation (Eq. 2) for an irreversible oxidation reaction was used to calculate the apparent diffusion coefficient (D_app_, cm^2^ L^-1^):^46^

(2) $$I_{p}^{irrev}=\pm 0.496{\left(\alpha \mathrm{n'}\right)}^{1/2}nFAC{\left(\frac{FDv}{RT}\right)}^{1/2}$$

Where *A* is the surface area (0.312 cm^2^), *α* is the transfer coefficient (taken as 0.5, on the basis of assuming that the ratio of oxidation peak current for reversible-to-irreversible is 1.27, that is 80% for irreversible processes compared to reversible one), *n* is the total number of electrons transferred per molecule in the electrochemical process, *nʹ* is the number of electrons transferred per mole before the rate determining step, *C* is the analyte concentration (1×10^−6^ mol cm^−3^), *F* is the faraday’s constant, R is the universal gas constant, *T* is the absolute temperature (T= 298 k), *D* is the diffusion coefficient (cm^2^/s) and *v* is the scan rate (V. L^-1^).^46^

Table 1 contained a summary of D_app_ values at different modified electrodes demonstrating highest value at CoGILCCP-SDS owing to the inherent catalytic activity and synergistic interactions of its individual modifiers and the role of SDS as preconcentrating agent of MO at the electrode surface. These values reflected the enhanced mass transfer of MO at CoGILCCP-SDS from solution bulk to electrode surface combining with fast electron transfer rate at electrode/solution interface.

#### Influence of supporting electrolyte pH

**Figure 4:**
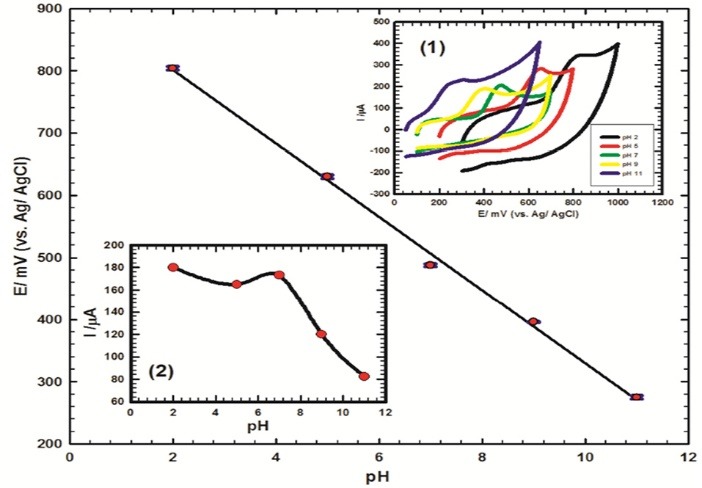
The linear relationship between the anodic peak potential of Morphine (MO) (mV) and the pH values (2-11). **Inset (1):** Cyclic voltammograms (CVs) of 1 mmol L^-1^ MO/0.1 mol L^-1^ phosphate buffer solution (PBS) with different pH values (2-11) at CoGILCCP-SDS. **Inset (2):** The relationship between the anodic peak current of MO (µA) and the pH values (2-11), scan rate 50 mV L^-1^.

The influence of supporting electrolyte pH on the electrochemical response of CoGILCCP-SDS electrode toward MO was studied. Figure 4 (Inset 1) showed the cyclic voltammograms of 1 mmol L^-1^ MO/0.1 mol L^-1^ PBS of different pH values (2-11) at CoGILCCP-SDS electrode. As the pH increased from 2 to 11, the oxidation potential of MO was shifted to less positive values and a linear relation was obtained between pH and oxidation potential (Figure 4). This study indicated that MO oxidation at CoGILCCP-SDS modified electrode is a pH dependent process, involving protonation/deprotonation steps. This linear relation could be fitted to (Eq. 3);

(3) Epa(V)=0.918−0.0588 pH(With  R2=0.997)

**Scheme 1:**
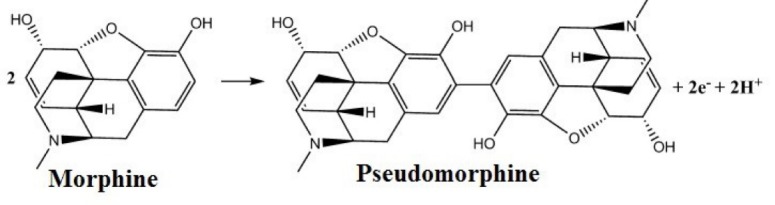
Electrochemical oxidation of morphine.

The slope of this relation is –58.8 mV/pH which is nearly the same as the Nernstian slope (–59 mV/pH) indicating that the electrochemical oxidation process of MO involves equal number of protons and electrons (1H^+^/1e^¯^) as shown in Scheme 1. The oxidation of MO involves the phenolic group oxidation to form pseudomorphine which possesses two phenolic groups. The oxidation peak at 478 mV includes the oxidation of MO and pseudomorphine.^12,14,15,17,18,41^

Figure 4 (Inset 2) showed the relation between the oxidation current of MO at CoGILCCP-SDS and pH showing the maximum current response at pH 7. So, pH 7.4 was chosen for the study to ensure the physiological conditions.

#### Sensor performance and method validation

#### Stability, reproducibility and repeatability

Supplementary file 4 showed the electrochemical response of 1 mmol L^-1^ MO/0.1 mol L^-1^ PBS/pH 7.4 at CoGILCCP-SDS upon repeating cycles up to 25 cycles. Stable signal was obtained with the same current response and oxidation potential indicating the fouling resistance of the proposed composite. In addition, repeatability and reproducibility are important parameters indicating the stability of the signal response. Repeatability was investigated by calculating the RSD of five runs of MO oxidation on the same CoGILCCP-SDS electrode. RSD was 0.96% demonstrating good repeatability of the proposed composite. Moreover, the reproducibility was investigated by calculating RSD of three runs at three similarly prepared CoGILCCP-SDS electrodes independently. RSD was 0.93% revealing good reproducibility of the proposed sensor.

#### Robustness

It is very crucial to study the stability of the current response at the proposed composite upon the impact of infinitesimal changes in the operational parameters. The parameters under investigation were Co_3_O_4_ content (5 % ± 0.1 % w/w), pH change (7.4 ± 0.2) and the time elapse before the measurement (2 min ± 2 s). The RSD values were 0.67%, 0.86% and 0.95%, respectively. The small values of RSD revealed the stability of the current response of MO at CoGILCCP-SDS upon minor changes in the experimental parameters and the robustness of the proposed method.^47^

#### Precision

The intra-day and inter-day precision values were investigated by the analysis of the same surface in the same concentration in a single run or three independent runs three times, respectively. The RSD values were 0.85% and 1.05%, respectively confirming that good precision was achieved using the proposed composite.^47^

#### Determination of morphine in real samples and tablets

#### Analysis of human urine samples

**Figure 5:**
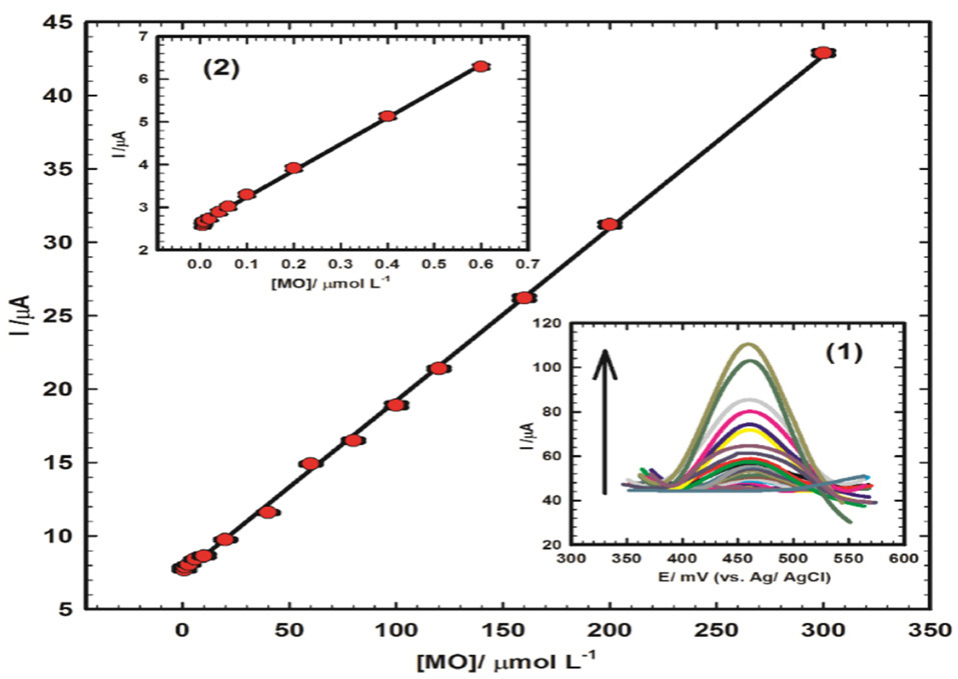
Calibration curve for morphine (MO) in the linear range (0.8 µmol L^-1^– 300 µmol L^-1^) at CoGILCCP-SDS. **Inset (1):** Differential pulse voltammograms (DPVs) of MO in urine at CoGILCCP-SDS for concentrations from (5 nmol L^-1^ to 300 µmol L^-1^). **Inset (2):** Calibration curve for MO in the linear range (5 nmol L^-1^ – 0.6 µmol L^-1^) at CoGILCCP-SDS.

The proposed composite, CoGILCCP-SDS, was examined for method validation according to technical reports of WHO.^47^ Direct determination of MO in urine samples as real samples and in pharmaceutical formulations was tested to confirm the validity of the proposed method for drug control analysis of MO. Figure 5 (Inset 1) showed the differential pulse voltammograms of standard additions of 1 mmol L^-1^ MO/0.1 mol L^-1^ PBS/pH 7.40 to 15 mL of diluted urine/pH 7.40 at CoGILCCP-SDS. Increasing the concentration of MO from 5 nmol L^−1^ to 300 μmol L^−1^, the anodic peak response of MO increased sharply. Figure 5 (Inset 2) showed the calibration curve in the linear range of 5 nmol L^−1^ to 0.6 μmol L^−1^ with the linear regression equation of (Eq. 4):

(4) $$I_{p}(\mu A)=6.19\;C\;(\mu mol\;L^{-1})+2.63$$

Figures of merit are: the correlation coefficient (R^2^) is 0.999, detection limit is 0.484 nmol L^−1^, quantification limit is 1.61 nmol L^−1^ and sensitivity is 6.19 µA/µmol L^-1^.

Figure 5 showed the calibration curve in the linear range of 0.8 μmol L^−1^ to 300 μmol L^−1^ with the linear regression equation of (Eq. 5):

(5) $$I_{p}(\mu A)=0.12\;C\;(\mu mol\;L^{-1})+7.48$$

Figures of merit are: the correlation coefficient (R^2^) is 0.999, detection limit is 2.554 nmol L^−1^, quantification limit is 8.153 nmol L^−1^ and sensitivity is 0.12 µA/µmol L^-1^.

The following equations were used to calculate detection (DL) and quantification limits (QL) (Eqs. 6 and 7, respectively):

(6) DL=3(s/b)

(7) QL=10(s/b)

Where “s” is the standard deviation and “b” is the slope of the calibration curve.

These results showed that MO can be determined sensitively in human urine with sub-nanomolar concentration and the proposed method was free from interferences present in urine matrix.

Table 3 contained a comparison for determination of MO at CoGILCCP-SDS with other modified electrodes mentioned in literature. Reasonable sensitivity, lower detection limit and wide linear range were the achievements of the proposed composite.

**Table 3:** Comparison for determination of morphine (MO) at various modified electrodes-based literature reports

**Electrode**	**Interfering agents**	**Linear dynamic range**	**Sensitivity/µA/µmol L** ^-1^	**Technique**	**Detection limit/nmol L** ^−1^
Chitosan coated Fe_3_O_4_ magnetic nano particle^12^	Na^+^, K^+^, Mg^2+^, Ca^2+^, Cl^−^, Li^+^, Al^3+^, NH^4+^, tryptophan, histidine, glycine, urea, dopamine, thiourea	10 nmol L^−1^ – 2 µmol L^−1^	Not reported	Differential pulse voltammetry	3
Gold nanoparticles ferrocene modified carbon paste electrode^14^	Ascorbic acid, uric acid, dopamine, norepinephrine	1.0 µmol L^−1^ –1800 µmol L^−1^	Not reported	Differential pulse voltammetry	3.5
Gold nanoparticles cobalt phthalocyanine modified carbon paste electrode^15^	Ascorbic acid, uric acid, dopamine, norepinephrine	0.4 µmol L^−1^– 900 µmol L^−1^	Not reported	Differential pulse voltammetry	5.48
Electrochemically reduced multiwall carbon nanotubes -doped graphene oxide glassy carbon electrode^16^	Uric acid, dopamine, codeine	0.07 nmol L^−1^– 6.5 µmol L^−1^	10.3	Linear sweep voltammetry	50
ZnO/carbon nanotubes/ ionic liquid modified carbon paste electrode^17^	Glucose, sucrose, lactose, fructose, K^+^, Li^+^, Ca^2+^, Mg^2+^, Al^3+^, NH^4+^, SO_4_^2−^, Cl^−^, ClO_4_^−^, methionine, alanine, phenylalanine, valine, tryptophan, histidine, glycine, starch, urea, thiourea	0.1 µmol L^−1^– 700 µmol L^−1^	Not reported	Linear sweep voltammetry	60
Graphene–palladium-hybrid-modified glassy carbon electrode^18^	Ascorbic acid, uric acid, dopamine	0.34 µmol L^−1^ – 12 µmol L^−1^	0.135	Differential pulse voltammetry	12.95
Multiwall carbon nanotubes modified carbon ionic liquid electrode^41^	Glucose, sucrose, lactose, fructose, citric acid, methanol, ethanol, Ca^2+^, Mg^2+^, SO_4_^2−^, Al^3+^, NH^4+^, alanine, phenylalanine, methionine, glycine, glutamic acid, tryptophan, urea, aspirin, thiourea, starch, cysteine, cystine, paracetamol, codeine	0.45 µmol L^−1^– 450 µmol L^−1^	0.025	Differential pulse voltammetry	140
CoGILCCP-SDS [This work]	Ascorbic acid, dopamine, terazosin	5 nmol L^−1^ – 0.6 µmol L^−1^	6.19	Differential pulse voltammetry	0.484

To investigate the accuracy and precision of the proposed method, four different concentrations were chosen to be repeated five times (Table 4). Acceptable recovery results in the range of 99.65% to 100.36% were obtained at CoGILCCP-SDS with RSD in the range of 0.071% to 2.133%. The obtained results were in good agreement with that obtained using other reported methods showing that this method achieved the validation for MO quality control analysis.^47^

**Table 4:** Evaluation of the accuracy and precision of the proposed method for the determination of morphine (MO) in urine samples

**Sample**	**[MO] added (µmol L** ^-1^ **)**	**[MO] found (µmol L** ^-1^ **)** ^a^	**Recovery (%)**	**Standard deviation ×10** ^-7^	**RSD** ^b^ ** (%)**	**Standard error** ^c^ **×10** ^-7^
1	0.005	0.0049	99.76	0.089	0.344	0.04
2	0.04	0.039	99.65	0.1732	0.599	0.077
3	0.6	0.599	99.96	0.04472	0.071	0.071
4	80	80.29	100.36	1.3416	2.133	0. 60

^a^ Average of five determinations. ^b^ Relative Standard deviation. ^c^ Standard error = Standard deviation/ n^1/2^.

#### Analysis of pharmaceutical tablets of morphine

Analysis of MO in its commercial tablets is necessary to examine the validity of CoGILCCP-SDS for pharmaceutical formulations analysis. Standard MO was injected into the electrochemical cell with a concentration of 2 µmol L^-1^. Then standard additions of MO tablets solution were introduced into the cell in the concentration range of (9 nmol L^-1^ – 100 µmol L^-1^). Equation (8) was used to calculate the analyzed sample concentration:

(8) $$\left[\text{Standard added (2 }\mu \text{mol }{\text{ L}}^{-1}\text{) + Tablet added (0.009-100 }\mu \text{mol }{\text{ L}}^{-1})\right]$$

Five concentrations were selected to be repeated five times and the recovery results were calculated and summarized in Table 5. Acceptable recovery results in the range of 99.99% – 101.81% were obtained with small RSD values. The proposed nanocomposite can be used for real sample analysis of MO in human urine and pharmaceutical tablets with excellent analytical performance.

**Table 5:** Evaluation of the accuracy and precision of the proposed method for the determination of morphine (MO) in pharmaceutical samples

**Tablet taken (µmol L** ^-1^ **)**	**Standard added (µmol L** ^-1^ **)**	**Found (µmol L** ^-1^ **)** ^a^	**Recovery (%)**	**Standard deviation × 10** ^-7^	**RSD** ^b^ ** (%)**	**SE** ^c^ ** × 10** ^-7^
0.009	2.00	2.0089	99.99	0.089	0.335	0.040
0.06	2. 00	2.0605	100.92	0.421	1.39	0.188
0.1	2.00	2.1003	100.30	0.742	2.25	0.332
10	2.00	12.031	100.31	0.493	0.515	0.577
100	2.00	101.81	101.81	5.77	3.30	3.33

^a^ Average of five determinations. ^b^ Relative Standard deviation. ^c^ Standard error = Standard deviation/ n^1/2^.

#### Simultaneous determination of morphine in presence of common interferences

**Figure 6:**
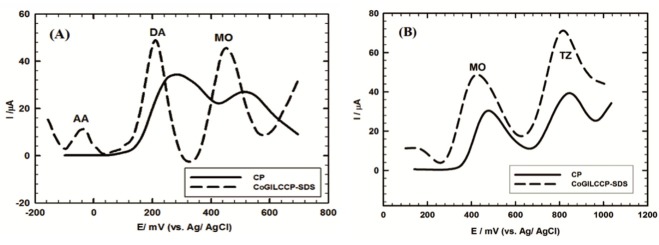
**(A)** Differential pulse voltammograms (DPVs) of 1 mmol L^-1^ morphine (MO) in the presence of 1 mmol L^-1^ ascorbic acid (AA) and 0.1 mmol L^-1^ dopamine (DA)/0.1 mol L^-1^ phosphate buffer solution (PBS)/pH 7.40 at CP and CoGILCCP-SDS. **(B)** DPVs of 1 mmol L^-1^ MO in the presence of 0.1 mmol L^-1^ terazosin (TZ)/0.1 mol L^-1^ PBS/pH 7.40 at CP and CoGILCCP-SDS.

It is very important from clinical point of view to examine the interference resistance of the proposed sensor toward the electrochemical determination of MO in presence of interferences present in the biological fluids like DA AA.^44,45^ Also, it is necessary to study the ability of the proposed sensor to discriminate MO from TZ for patients under terazosin treatment. Figure 6A showed the DPVs of a tertiary mixture of 1 mmol L^-1^ AA, 0.1 mmol L^-1^ DA and 1 mmol L^-1^ MO/0.1 mol L^-1^ PBS/pH 7.4 at CP and CoGILCCP-SDS electrodes. Overlapped and unresolved two peaks were obtained at CP while three well-defined and separated peaks with higher current response were obtained at CoGILCCP-SDS at –36 mV, 212 mV and 452 mV for AA, DA and MO, respectively. Besides, the simultaneous determination of a binary mixture of 1 mmol L^-1^ MO and 0.1 mmol L^-1^ TZ/0.1 mol L^1^ PBS/pH 7.4 was achieved at CP and CoGILCCP-SDS as shown in Figure 6B. Two well-resolved anodic peaks were obtained at CoGILCCP-SDS at 422 mV and 816 mV for MO and TZ, respectively with higher current response compared to CP. These results revealed the capability of the proposed composite to simultaneously detect MO in presence of interfering compounds with high current response and excellent potential separation.

**Figure 7:**
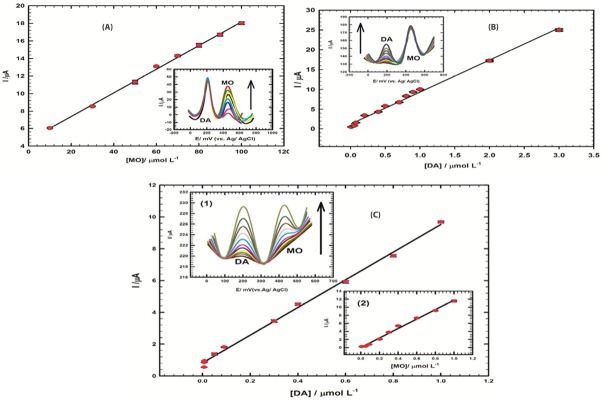
**(A)** Calibration curve for morphine (MO) in the presence of 0.1 mmol L^-1^ dopamine (DA) in the linear range (10 µmol L^-1^– 100 µmol L^-1^) at CoGILCCP-SDS. **Inset:** Differential pulse voltammograms (DPVs) of MO in the presence of 0.1 mmol L^-1^ DA at CoGILCCP-SDS for concentrations from 10 µmol L^-1^ to 100 µmol L^-1^. **(B)** Calibration curve for DA in the presence of 1 mmol L^-1^ MO in the linear range (0.01 µmol L^-1^– 3 µmol L^-1^) at CoGILCCP-SDS. **Inset:** DPVs of DA in the presence of 1 mmol L^-1^ MO at CoGILCCP-SDS for concentrations from 0.01 µmol L^-1^ to 3 µmol L^-1^. **(C)** Calibration curve for DA in the linear range (0.006 µmol L^-1^– 1 µmol L^-1^). **Inset (1):** DPVs of MO in the presence of DA at CoGILCCP-SDS for concentration ranges (0.008 µmol L^-1^– 1µmol L^-1^) of MO and (0.006µmol L^-1^ – 1µmol L^-1^) of DA at CoGILCCP-SDS. **Inset (2):** Calibration curve for MO in the linear range (0.008µmol L^-1^ – 1µmol L^-1^) at CoGILCCP-SDS.

Moreover, the simultaneous determination of MO in the concentration range of 10→100 µmol L^−1^ was studied at CoGILCCP-SDS in presence of constant concentration of 0.1 mmol L^−1^ DA as shown in Figure 7A (Inset). The calibration curve of MO in the concertation range of 10→100 µmol L^−1^ in presence of 0.1 mmol L^−1^ DA was shown in Figure 7A with the linear regression equation of (Eq. 9):

(9) $$I_{p}(\mu A)=0.135\;C\;(\mu mol\;L^{-1})+4.70$$

The correlation coefficient is: R^2^ = 0.998.

The detection limit of MO was 1.3 nmol L^-1^ and the sensitivity was 0.135 µA/µmol L^-1^.

Also, the DPVs of DA in the concentration range of 0.01 – 3 µmol L^−1^ in presence of 1 mmol L^−1^ MO were shown in Figure 7B (Inset). The calibration curve for DA in the concentration range of 0.01 – 3 µmol L^−1^ in presence of 1 mmol L^−1^ MO was obtained at CoGILCCP-SDS as shown in Figure 7B with the linear regression equation of (Eq. 10):

(10) $$\begin{array}{l} I_{p}(\mu A)=8.08\;C\;(\mu mol\;L^{-1})+1.27,\\ \left(\text{With }{\text{ R}}^{2}=0.993\right) \end{array}$$

The sensitivity and detection limit were 8.08 μA/µmol L^−1^ and 0.37 nmol L^−1^, respectively for DA. Further study was done to examine the sensitivity of CoGILCCP-SDS sensor for the simultaneous determination of low concentration levels of MO and DA mixture. Figure 7C (Inset 1) showed simultaneous determination of MO and DA by using DPV and the plot of the peak current vs. MO concentration was linear for the concentration range of (0.008 – 1 µmol L^−1^) Figure 7C  (Inset 2), with a regression equation of (Eq. 11):

(11) $$\begin{array}{l} I_{p}(\mu A)=11.73\;C\;(\mu mol\;L^{-1})+0.01,\\ \left(\text{With }{\text{ R}}^{2}=0.993\right) \end{array}$$

While, the regression equation for DA in the linear concentration range of (0.006 – 1 µmol L^−1^) was:

(12) $$\begin{array}{l} I_{p}(\mu A)=8.7\;C\;(\mu mol\;L^{-1})+0.83,\\ \left(\text{With }{\text{R}}^{2}=0.997\right) \end{array}$$

The sensitivities and detection limits were 11.73μA/µmol L^-1^ and 0.54 nmol L^−1^ for MO and 8.70 μA/µmol L^-1^ and 0.25 nmol L^−1^ for DA, respectively.

The previous result confirmed the capability of CoGILCCP-SDS to sensitively and simultaneously detect MO and DA in low concentration levels demonstrating the featured analytical performance of the proposed composite.

## Conclusion

In the present study, an electrochemical sensor CoGILCCP-SDS was fabricated for the sensitive nanomolar determination of morphine in human urine and pharmaceutical tablets. The sensor was based on good synergism between cobalt oxide nanoparticles, graphene and ILC modified CP electrode in presence of SDS; CoGILCCP-SDS. This nanocomposite sensor exhibited excellent conductivity and catalytic capability in enhanced electron transfer kinetics compared to other studied electrodes. Moreover, simultaneous determination of low concentration levels for MO and DA mixture was achieved with low detection limits of 0.54 nmol L^−1^ and 0.25 nmol L^−1^, respectively. The characteristic features of the proposed sensor was anti-interference capability toward MO in presence of interfering species; AA, DA or TZ. The proposed sensor was applied for determination of MO in urine sample in the linear dynamic range of 5 nmol L^-1^ to 0.6 μmol L^−1^ with detection limit of 0.484 nmol L^−1^, quantification limit of 1.61 nmol L^−1^ and sensitivity of 6.19 µA/µmol L^-1^. The proposed sensor presented good characteristics which further extended its application for electrochemical determination of other narcotics in human fluids with good sensitivities and nanomolar detection limits.

## Ethical Issues

Not applicable.

## Conflict of Interest

There is no conflict of interest to declare.

## Acknowledgments

The authors appreciated the financial support from Cairo University through the Office of the President for Research Funds.

## Supplementary Materials


**Supplementary file 1.** CVs of 1 mmol L^-1^ MO/0.1 mol L^-1^ PBS/pH 7.40 at CP, CoILCCP CoGCP, GILCCP, CoILCCP-SDS, CoGCP-SDS, GILCCP-SDS, and CoGILCCP-SDS.

**Supplementary file 2.** CVs of 1 mmol L^-1^ MO/0.1 mol L^-1^ PBS/pH 7.40 at CoGILCCP-SDS, CoGIL1CP-SDS and CoGIL2CP-SDS, scan rate 50 mV L^-1^.

**Supplementary file 3.** Relationship between the anodic peak current of MO (µA) and the square root of the scan rate (V L^-1^)^1/2^. **Inset:** CVs of 1 mmol L^-1^ MO/0.1 mol L^-1^ PBS/pH 7.40 at CoGILCCP-SDS at different scan rates (10–100 mV L^-1^).

**Supplementary file 4.** Repeated cycles stability of 1 mmol L^-1^ MO/0.1 mol L^-1^ PBS/pH 7.40 at CoGILCCP-SDS, 25 repeated cycles, scan rate 50 mV L^-1^.
