# Supplementary material for: Cobalt Oxide Nanoparticles/Graphene/Ionic Liquid Crystal Modified Carbon Paste Electrochemical Sensor for Ultra-sensitive Determination of a Narcotic Drug
Source: Adv Pharm Bull. 2018 Feb 21;9(1):110–21. doi: 10.15171/apb.2019.014 (PMC6468225; doi:10.15171/apb.2019.014)

Supplementary file 1. CVs of 1 mmol L^{-1} MO/0.1 mol L^{-1} PBS/pH 7.40 at CP, CoILCCP CoGCP, GILCCP, CoILCCP-SDS, CoGCP-SDS, GILCCP-SDS, and CoGILCCP-SDS.

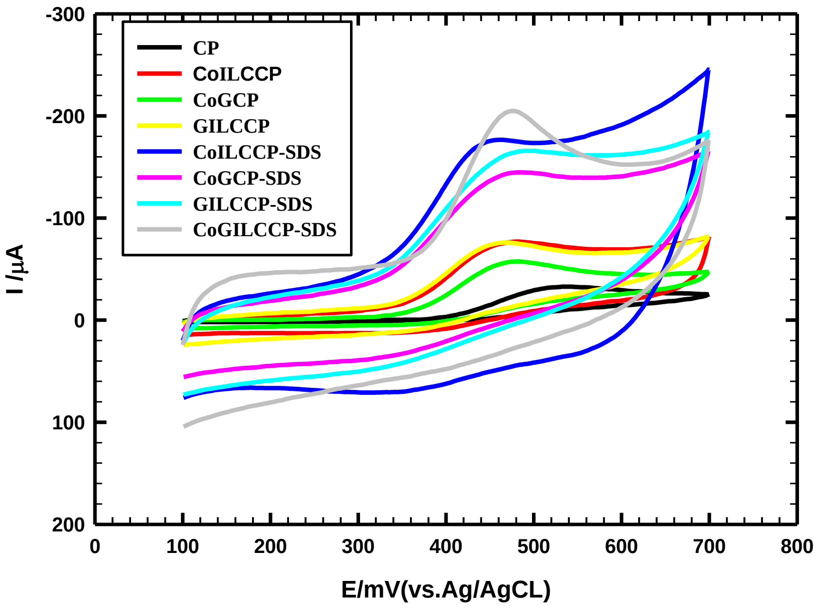

Supplement: Supplementary file 1 — CVs of 1 mmol L-1 MO/0.1 mol L-1 PBS/pH 7.40 at CP, CoILCCP CoGCP, GILCCP, CoILCCP-SDS, CoGCP-SDS, GILCCP-SDS, and CoGILCCP-SDS. [file apb-9-110-s001.pdf]
