# Supplementary material for: Cobalt Oxide Nanoparticles/Graphene/Ionic Liquid Crystal Modified Carbon Paste Electrochemical Sensor for Ultra-sensitive Determination of a Narcotic Drug
Source: Adv Pharm Bull. 2018 Feb 21;9(1):110–21. doi: 10.15171/apb.2019.014 (PMC6468225; doi:10.15171/apb.2019.014)

Supplementary file 3. Relationship between the anodic peak current of MO (µA) and the square root of the scan rate (V $s^{-1})^{1/2}$. **Inset:** CVs of 1 mmol L^{-1} MO/0.1 mol L^{-1} PBS/pH 7.40 at CoGILCCP-SDS at different scan rates (10–100 mV s^{-1}).

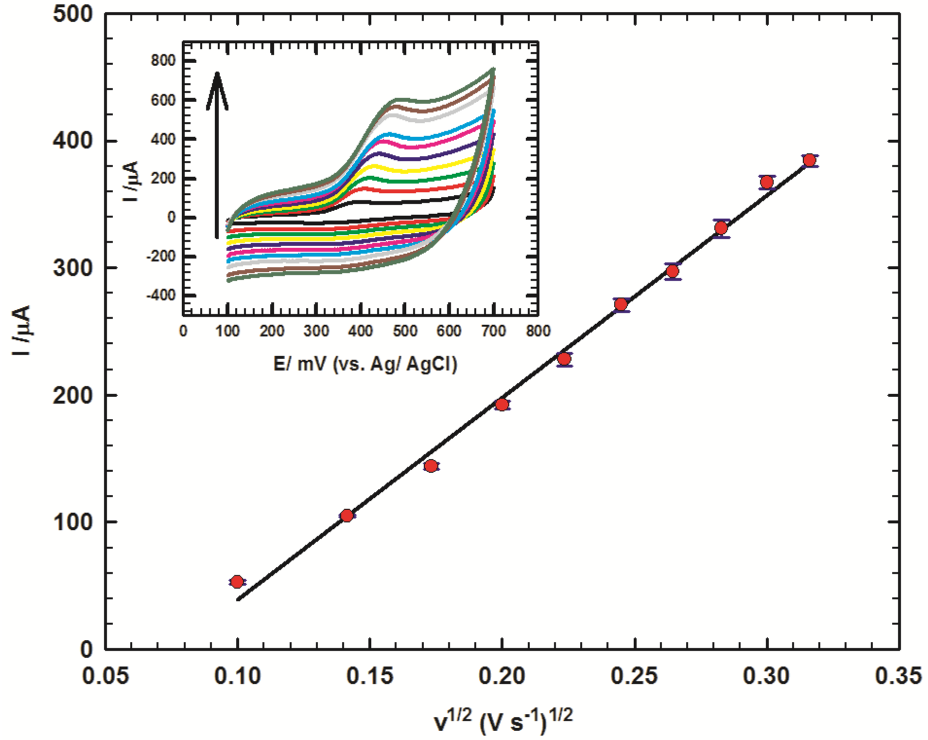

Supplement: Supplementary file 3 — Relationship between the anodic peak current of MO (µA) and the square root of the scan rate (V L-1)1/2. Inset: CVs of 1 mmol L-1 MO/0.1 mol L-1 PBS/pH 7.40 at CoGILCCP-SDS at different scan rates (10–100 mV L-1). [file apb-9-110-s003.pdf]
