# Supplementary material for: Cobalt Oxide Nanoparticles/Graphene/Ionic Liquid Crystal Modified Carbon Paste Electrochemical Sensor for Ultra-sensitive Determination of a Narcotic Drug
Source: Adv Pharm Bull. 2018 Feb 21;9(1):110–21. doi: 10.15171/apb.2019.014 (PMC6468225; doi:10.15171/apb.2019.014)

Supplementary file 4. Repeated cycles stability of 1 mmol L^{-1} MO/0.1 mol L^{-1} PBS/pH 7.40 at CoGILCCP-SDS, 25 repeated cycles, scan rate 50 mV s^{-1}.

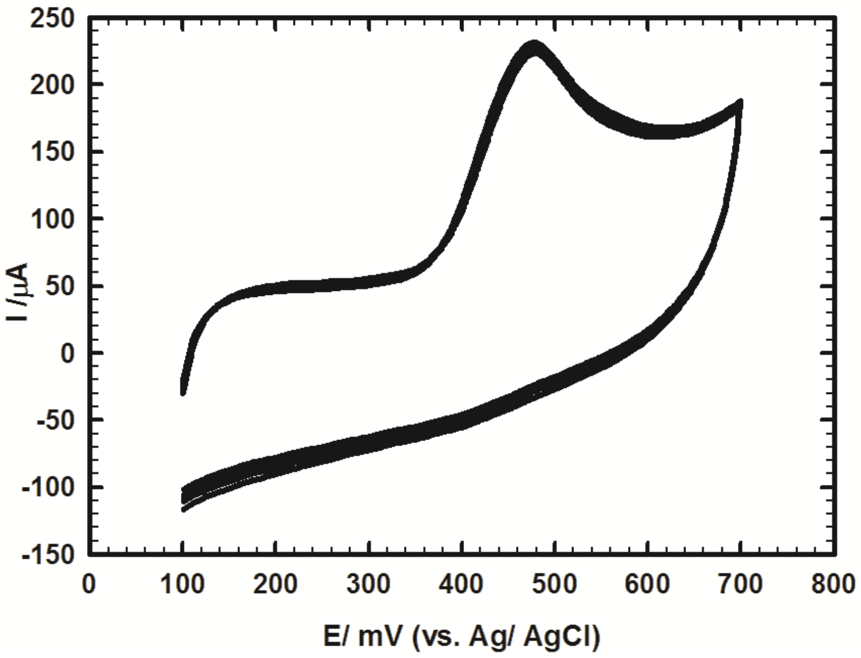

Supplement: Supplementary file 4 — Repeated cycles stability of 1 mmol L-1 MO/0.1 mol L-1 PBS/pH 7.40 at CoGILCCP-SDS, 25 repeated cycles, scan rate 50 mV L-1. [file apb-9-110-s004.pdf]
